# OA06 A complex case of thigh pain in Granulomatosis with Polyangiitis (GPA)

**DOI:** 10.1093/rap/rkac066.006

**Published:** 2022-09-28

**Authors:** Gen Nen Ho, Kishaani Suseeharan, Akshay Sachdev, Rameez Arif, Holly John

**Affiliations:** Dudley Group NHS Foundation Trust, Dudley, United Kingdom; Walsall Healthcare NHS Trust, Walsall, United Kingdom; Dudley Group NHS Foundation Trust, Dudley, United Kingdom; Dudley Group NHS Foundation Trust, Dudley, United Kingdom; Dudley Group NHS Foundation Trust, Dudley, United Kingdom

## Abstract

**Introduction/Background:**

Rheumatic pain can result from a multitude of aetiologies. We present a complex case of thigh pain as the presenting manifestation of GPA, which required a multi-disciplinary approach to investigate, diagnose and manage. The pain was attributed to lumbosacral plexopathy, a rare neurological manifestation of GPA. The case was further complicated by subsequent leg weakness secondary to steroid-induced myopathy underlining the challenges confronting clinicians in diagnosing and managing complex systemic conditions.

**Description/Method:**

A 73-year-old male was admitted with severe crampy bilateral thigh and buttock pain associated with reduced exercise tolerance. He had been well, other than a past history of bladder cancer, until 4 weeks previously when he developed a prodrome, sinusitis and epistaxis. Bloods showed raised inflammatory markers, a strongly positive PR3 and a normal CK. On examination he had no synovitis and no vasculitic rash. There was tenderness around the hamstrings and ischial tuberosities. Neurological examination showed no evidence of motor weakness or sensory disturbance. Urine dip showed +1 protein and +1 blood with normal ACR.

CT thorax showed no lung involvement. MRI head showed mild mucosal thickening in the ethmoid air cells. Nasal biopsy was inadequate, only showing granulation tissue. MRI spine showed L4/5 central canal narrowing with impingement of traversing bilateral L5 nerve roots. Nerve conduction studies and EMG showed multiple nerves with reduced amplitude on motor and sensory studies, consistent with a bilateral lumbosacral plexopathy.

He was treated as systemic GPA with high dose steroids and a plan for rituximab. The patient declined rituximab over Christmas so had methylprednisolone pulses before receiving rituximab.

Two weeks later he developed new proximal leg weakness, power 2/5. Repeat MRI spine was unchanged and CK was normal. ANA was weakly positive with a negative myositis blot including Jo-1. MRI thighs showed bilateral symmetrical muscle changes suggestive of acute myositis, intriguingly with onset whilst on high dose steroids. Clinical impression was a steroid myopathy; steroids were weaned and the weakness improved.

Eight weeks after rituximab he had significantly improved, with resolution of epistaxis and severe thigh pain and much improved exercise tolerance, as well as inflammatory markers settling and almost normalisation of his PR3 titre. Repeat MRI thighs after a 3-month interval showed a significant reduction in muscle oedema.

**Discussion/Results:**

The thigh pain was challenging to diagnose. It is anatomically distinct from other peripheral nervous system manifestations reported in GPA, such as distal symmetrical sensory neuropathy (7-10% of GPA), or motor mononeuritis multiplex (2-12% of GPA). However, the pieces fell into place when nerve conduction study confirmed lumbosacral plexopathy. Existing literature suggests that lumbosacral plexopathy typically presents as lower back pain radiating to the leg, often worse in supine position and may be associated with abnormal sensation and weakness in the lower limbs, in keeping with the nature of his thigh pain.

Albeit rare, lumbosacral plexopathy has been associated with inflammatory diseases e.g. sarcoidosis, but there is little reporting of lumbosacral plexopathy in GPA specifically. Brachial plexopathy has been reported in microscopic polyangiitis; they proposed that vasculitic arterial ischaemia leads to plexus ischaemia. Wider differentials of plexopathy include diabetic amyotrophy, paraneoplastic phenomenon, autoimmune (including CIDP), localised trauma or infection; these should be ruled out with relevant investigations.

His vasculitic ENT symptoms settled with initial high dose steroids, but the excruciating thigh pain did not. When he developed thigh weakness having just received pulsed methylprednisolone, MRI thigh showed changes suggestive of acute myositis, which added another layer of complexity. Normal CK and negative myositis autoantibodies made new-onset myositis unlikely. The multi-specialty consensus was steroid-induced myopathy, which retrospectively seems most plausible as his symptoms improved with steroid reduction, as did interval MRI follow-up scan.

MRI, while highly sensitive for detecting muscle disorders, has limited specificity in distinguishing the type of myopathy and therefore is rarely performed in clinical practice to diagnose steroid-induced myopathy. MRI findings must be correlated with the clinical context. If in doubt, a muscle biopsy should be sought. This would form our next line of investigation if his thigh symptoms had not settled.

**Key learning points/Conclusion:**

The International Association for the Study of Pain (IASP) has recently defined pain as ‘’an unpleasant sensory and emotional experience associated with, or resembling that associated with, actual or potential tissue damage’’. Acute causes of pain are varied and include local and systemic aetiologies such as inflammatory, infective, traumatic, malignant and iatrogenic.

At presentation, the patient’s main symptom was “excruciating severe cramping pain”. This case emphasizes the importance of a thorough clinical approach comprising detailed history and examination which guided focussed investigations and discussions with specialised multi-disciplinary teams (rheumatology, ENT, radiology, neurology, neurophysiology) to unravel the complexity of this clinical scenario.

Whilst peripheral nervous system involvement, most frequently mononeuritis multiplex, is common in ANCA-associated vasculitis, plexopathy is not; however, discussion with the neurophysiologist was extremely helpful in interpreting complex neurophysiology tests to appreciate it was a vasculitic manifestation.

As symptoms evolve, always consider if they reflect the underlying disease or are sequelae of treatment. The likelihood of developing acute myositis whilst on high dose steroids was less plausible than a steroid myopathy; always treat the patient and the clinical scenario.

Interval scanning can be helpful to monitor progress and response to treatment; in this case it was reassuring that repeat MRI thigh showed a reduction in muscle oedema once steroids were decreased.

Whilst “common things are common”, rare presentations of rare conditions also occur; utilise the expertise of your MDT in these situations.

